# The secret life of Pickering emulsions: particle exchange revealed using two colours of particle

**DOI:** 10.1038/srep31401

**Published:** 2016-08-10

**Authors:** David J. French, Aidan T. Brown, Andrew B. Schofield, Jeff Fowler, Phil Taylor, Paul S. Clegg

**Affiliations:** 1 School of Physics & Astronomy, The University of Edinburgh, Peter Guthrie Tait Road, Edinburgh, EH9 3FD, UK; 2 Syngenta Inc., 410 Swing Rd, P.O.Box 183000, Greensboro, North Carolina, 27419-8300, USA; 3 Formulation Technology Group, Syngenta Crop Sciences, Jealott’s Hill International Research Centre, Bracknell, Berkshire, RG42 6EY, UK

## Abstract

Emulsion droplets stabilised by colloidal particles (Pickering emulsions) can be highly stable, so it is unsurprising that they are beginning to be exploited industrially. The individual colloidal particles have interfacial attachment energies that are vastly larger than the thermal energy, hence they are usually thought of as being irreversibly adsorbed. Here we show, for the first time, particles being exchanged between droplets in a Pickering emulsion. This occurs when the emulsion contains droplets that share particles, often called bridging. By starting with two emulsions showing bridging, each stabilised by a different colour of particle, the dynamics can be studied as they are gently mixed together on a roller bank. We find that particle exchange occurs by two routes: firstly, during a period of unbridging and rebridging whose duration can be tuned by varying the wettability of the particles, and secondly, during very rare events when particles are ejected from one droplet and re-adsorbed onto another.

Formulations based on natural ingredients are currently in great demand, making it highly desirable to replace synthetic small-molecule surfactants with benign colloidal particles in emulsion-based products. The resulting Pickering emulsions are usually considered to be very stable, due to the large reduction in free energy, Δ*G*, occurring when a particle moves from its preferred phase to the oil-water interface. This can be expressed as


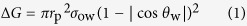


where *θ*_w_ is the equilibrium three-phase contact angle, *σ*_ow_ is the interfacial tension, and *r*_p_ is the particle radius^1^. A particle with radius 0.5 *μ*m and a contact angle of 90° at a typical oil-water interface (*σ*_ow_ ≈ 50 mN m^−1^) will have a trapping energy of approximately 10^6^ *k*_B_*T* (where *k*_B_ is Boltzmann’s constant and *T* is the absolute temperature). Even when *θ*_w_ = 30°, Δ*G* ≈ 10^4^ *k*_B_*T*. This is in stark contrast to small-molecule surfactants, where Δ*G* ≈ 5*k*_B_*T*, which tend to ‘hop’ on and off the interface. It is therefore tempting to conclude that the particles are irreversibly adsorbed at the oil-water interface^1^,^2^,^3^,^4^,^5^,^6^,^7^,^8^,^9^. Indeed, irreversible adsorption has the status of the standard dogma of the field.

It is possible for droplets in a Pickering emulsion to share particles, as shown in Fig. 1(a–d), a phenomenon known as bridging^10^,^11^,^12^,^13^. If these particles, which are shared by two droplets, are irreversibly adsorbed to both interfaces then bridge breaking should not occur at any modest shear rate. However, as shown in ref. 13, particle bridges are actually broken by low shear, and this can even lead to free silica particles being released into the (continuous) aqueous phase, showing that particle adsorption is not irreversible. The release of silica particles into the continuous phase is presumably the result of relatively small forces generating relatively large torques in a manner reminiscent of the shearing of dendritic particle branches by gravitational forces^14^, although coalescence events between droplets and any free oil might also release silica particles into the aqueous phase.

Here we deploy a novel technique that involves the use of two batches of particles, which have been fluorescently labelled with different dyes, to study the degree to which a Pickering emulsion is remixed during low shear, both qualitatively and quantitatively. A preliminary attempt to search for particle bridging using this approach was unsuccessful^15^. Additionally, we note that this method could also be used to probe the fluidity of the particle layer. A conceptually related technique was developed by Taisne *et al*. to study coalescence events in oil-in-water emulsions produced using a high-pressure homogeniser^16^. In that work, two emulsions were created with oil phases of differing refractive index, so that they became gradually less opaque as coalescence progressed. In our work, we use two emulsions stabilised by different colours of particles. Both the average colour of our droplet surfaces, and the arrangement of the different colours on each droplet, tell us about the history of the droplets.

Our model emulsion system is made up of oil droplets in water with the interfaces stabilised using colloidal silica particles. The oil phase is a blend of dodecane and isopropyl myristate. Increasing the concentration of the isopropyl myristate makes the oil phase more polar; this tends to pull the hydrophilic particles further into the oil phase^17^. This is shown by the wetting angle at the particle surfaces, which becomes higher as the volume fraction of isopropyl myristate in the oil phase, Φ_IM_, increases^13^. Additionally, our experiments follow the detailed behaviour of the emulsions over long periods of time. We find that there is a slight drift in the wetting angle of the particles towards becoming more hydrophilic over time, due to water adsorbing at the surface of the particles^18^.

In this article we show that when two bridged Pickering emulsions are combined and gently stirred they steadily exchange their interfacial particles. We demonstrate this via the use of two different colours of particles, as described above. It rapidly becomes evident that the behaviour is not due to droplet coalescence, but is mainly caused by particles transferring between droplets during an unbridging/rebridging process. We then go on to modify the wetting properties of the particles (via a change to the composition of the oil phase) so as to weaken the bridges between droplets. This leads to the clusters of droplets becoming mixed more quickly. Via the addition of salt, we then completely suppress bridge formation; this reduces the amount of particle exchange without preventing it entirely; particle exchange in this case is primarily via a second process, where particles transferred are first released into the continuous phase and are then re-adsorbed onto a droplet’s surface. This second process is vastly slower than the unbridging/rebridging route. Because the unbridging/rebridging process can be suppressed via the controlled breaking of bridges (for example, via a sudden change in pH), the particle exchange behaviour is a readily tunable feature of Pickering emulsions.

## Results

### Particle exchange between bridged emulsions

**Figure 1:**
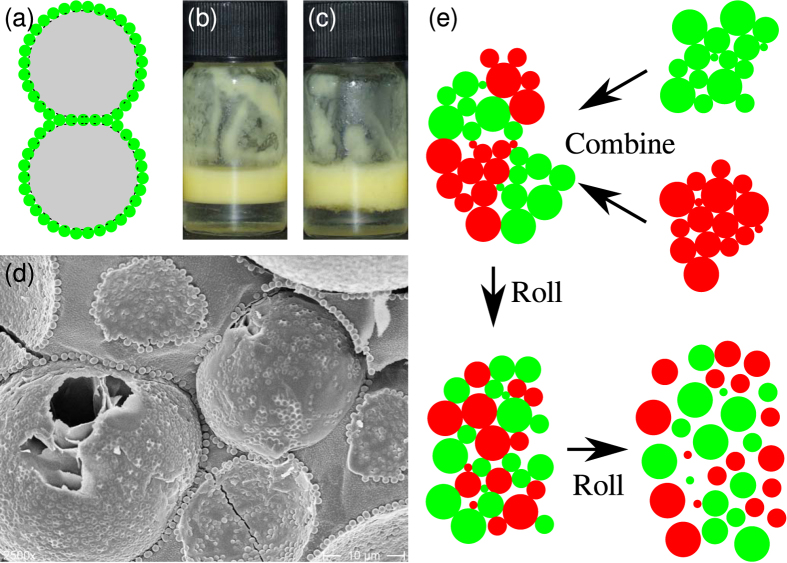
Bridging and particle transfer at schematic and practical levels. (**a**) Cartoon showing two emulsion droplets sharing particles, a phenomenon known as bridging. (**b**,**c**) The differences in identical composition emulsions without and with bridges, respectively. The sample containing bridges has a rough emulsion-aqueous phase interface, and the cream layer does not pack as closely. (**d**) Freeze-fracture scanning electron micrograph of emulsion droplets sharing particles. (**e**) Cartoon showing the disaggregation of a bridged two-colour sample.

**Figure 2:**
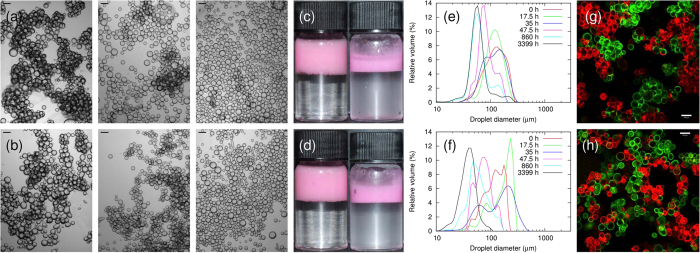
Evolution of two emulsions with different particle contact angles. (**a**,**b**) Micrographs of samples at various rolling times. (**a**) Sample with *ϕ*_p_ = 0.6% and Φ_IM_ = 50%; the rolling times are, from left to right, 1 h, 185 h and 860 h. (**b**) Sample with *ϕ*_p_ = 0.6% and Φ_IM_ = 10%; the rolling times are, from left to right, 1 h, 65 h and 185 h; Scale bars are 100 *μ*m. (**c**,**d**) Photographs of samples with (**c**) Φ_IM_ = 50% and (**d**) 10% at two rolling times. The rolling times are 0 h (left column) and 357 h (right column). (**e**,**f**) Apparent size distributions of the sample with *ϕ*_p_ = 0.6% and (**e**) Φ_IM_ = 50% or (**f**) Φ_IM_ = 10% at various rolling times. (**g**,**h**) Confocal micrographs of samples with Φ_IM_ values of (**g**) 50% and (**h**) 10%, after being on a roller bank for 3.5 h. The clusters in the sample with Φ_IM_ = 10% are more thoroughly mixed than those in the sample with Φ_IM_ = 50%; the scale bars are 100 *μ*m.

In a typical experiment, two bridged precursor emulsions which are identical apart from particle colour (composition: *ϕ*_p_ = 0.6%, Φ_IM_ = 50%, see Methods) were combined and then placed on a roller bank. As shown in Fig. 2(a), the bridging makes the droplets appear aggregated. The shear rate due to the roller bank is low (circumferential velocity divided by the diameter of the vial gives 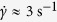) so droplet breakup is not expected to occur^19^. However, the emulsion clearly changes due to the rolling, as shown in micrographs (Fig. 2(a)) and photographs (Fig. 2(c)). Over a period of days, the gentle rolling causes the emulsion to gradually disaggregate as the bridges between droplets are broken, as shown schematically in Fig. 1(e). By itself, this indicates that the particles cannot be irreversibly adsorbed to every droplet.

The distributions of the sizes of droplets/aggregates, as measured using light scattering, are shown for various rolling times in Fig. 2(e). After two days of rolling, a peak at the droplet size emerges from the broader peak corresponding to the aggregate size. Although a layer of free oil develops and grows in volume as the experiment proceeds, there is still a significant dispersed volume even after several months of rolling. The layer of free oil is never large enough to be visible in photographs of the samples. The loss of sample volume observed in Fig. 1(c,d) is due to repeated sampling for imaging and size distribution studies; the volume of the emulsion cream also decreases as the degree of bridging reduces, because the emulsion droplets can pack more closely^13^.

**Figure 3:**
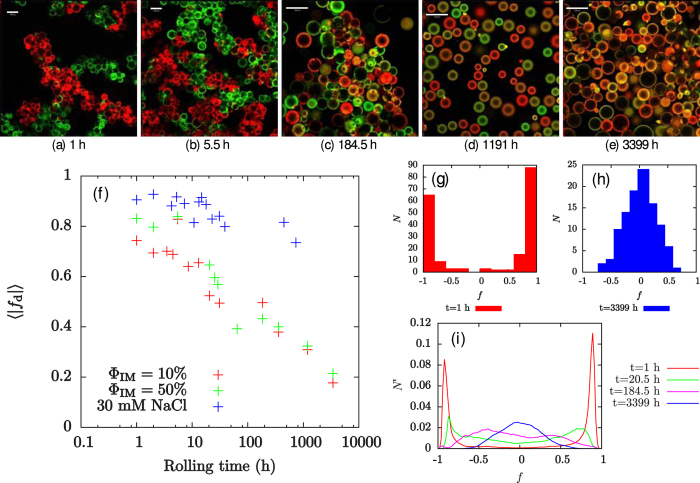
Tracking exchange using two colours of particles. (**a**–**e**) Confocal micrographs show that the clusters become more mixed as the sample is rolled. Simultaneously, the individual droplets become more heterogeneous; the scale bars are 100 *μ*m. (**f**) The value of 〈| *f*_d_|〉 decreases with rolling time, with the rate of decrease being greater in samples containing particle bridges. (**g**,**h**) Histograms of *f* for the sample with Φ_IM_ = 50% at two different rolling times. (**i**) Normalised plots of *f* at various rolling times for the sample with Φ_IM_ = 50%.

**Figure 4:**
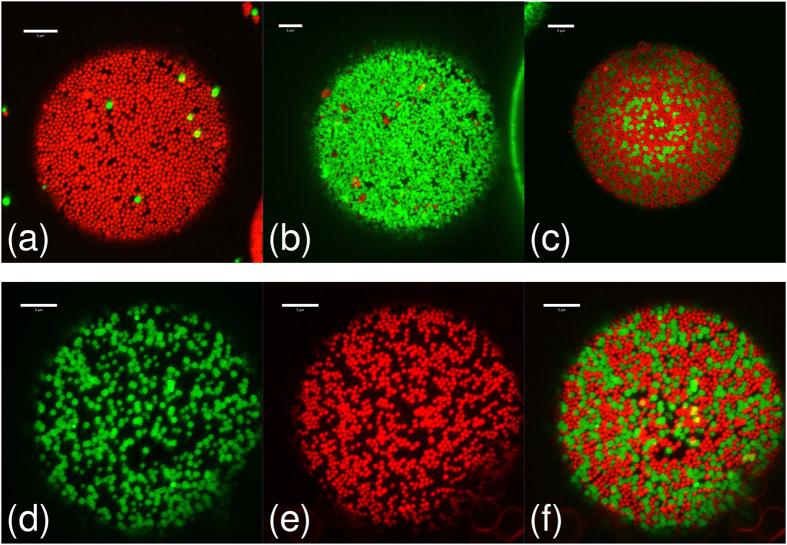
Droplet interface details. High magnification confocal micrographs of the Φ_IM_ = 50% sample from Fig. 3(a–e) showing the particle monolayers at droplet interfaces at various rolling times. The rolling times are (**a**) 0 h, (**b**) 5.5 h, (**c**) 22 h and (**d**–**f**) 357 h. Images (**d**–**f**) are of the same droplet, showing (**d**) the green channel, (**e**) the red channel and (**f**) the merged image. Each image is a z-projection of 10 images, with a z-spacing of 0.42 *μ*m, constructed using ImageJ^28^. All scale bars are 5 *μ*m.

The use of fluorescently labelled particles allows us to study the dynamics of the disaggregation process via confocal microscopy both qualitatively and quantitatively (see Methods), and to deduce whether particle bridges ever re-form after they have broken. Figure 3(a–e) shows a series of confocal micrographs from a single sample and Fig. 4 shows high magnification confocal micrographs of individual droplets from the same sample. At low rolling times, each cluster in the sample has a single colour, indicating that they are not mixed (see Fig. 3(a,g)). However, as the rolling time is increased, the colour of the clusters of droplets becomes steadily more mixed, even while the sample remains strongly bridged (see Fig. 3(b,c)). This shows that particle bridges are being broken and re-formed during the rolling process.

**Figure 5:**
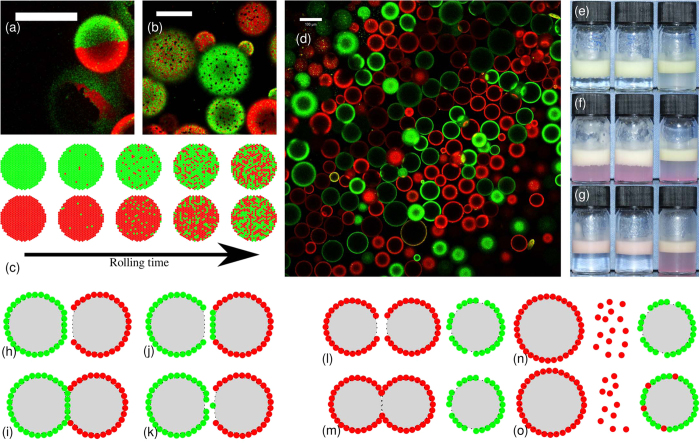
Mixing on the droplet interfaces. (**a**) Image of the Φ_IM_ = 10% sample after rolling for 13 h showing the detail of a Janus droplet; the scale bar is 50 *μ*m. (**b**) Φ_IM_ = 10% sample following 185 h of rolling showing the detail of bare patches (dark spots) which are thought to be caused by particle transfer; the scale bar is 50 *μ*m. (**c**) Cartoon showing particle transfer in two-colour samples. (**d**) Confocal micrograph of a sample containing 30 mM sodium chloride, following 280 h of rolling; the scale bar is 100 *μ*m. (**e**–**g**) Photographs showing free silica particles adsorbing to the oil-water interface in emulsions. In each photograph, the sample on the left has Φ_IM_ = 10%, the centre sample has Φ_IM_ = 50% and the sample on the right has Φ_IM_ = 10% and an aqueous NaCl concentration of 30 mM. (**e**) The samples following emulsification. (**f**) The samples following addition of the extra particles. (**g**) The samples following 1 h of rolling. (**h**–**k**) Cartoons showing two possible routes for droplet interfaces to become heterogeneous during disaggregation of a bridged Pickering emulsion. (**h**–**i**) A fully coated droplet collides with a partially coated droplet, forming a particle bridge. (**j**–**k**) The particle bridge is broken, leading to a patchy droplet or single particle transfer. (**l**–**o**) Cartoons showing a tentative route for particle re-adsorption. Two partially coated droplets coalesce, leading to the release of some particles into the continuous phase. These particles are then able to adsorb to the interface of a third partially coated droplet.

The mixing effect goes beyond the clusters of droplets: the individual droplets become steadily more heterogeneous, as shown in Fig. 3(f–i) and in Fig. 4. Figure 3(a,g) shows that each droplet initially contains only one colour of particle. However, as the rolling time is increased, the droplet interfaces become more heterogeneous until eventually it is impossible to discern whether a droplet was initially stabilised by FITC- or RITC-labelled particles, as shown in Fig. 3(e,h). Occasional droplets are observed to have Janus-like particle distributions (see Fig. 5(a)), indicating that coalescence has taken place, but coalescence cannot account for the gradual changes in droplet colour which are observed at fixed droplet size. These rare coalescence events may well occur during slide preparation.

Free silica particles are observed in the aqueous phase of the samples, becoming especially noticeable once disaggregation is complete. We have previously reported the presence of very large droplets when a bridged emulsion is sheared at a shear rate which is lower than that necessary to cause droplet breakup (but at a shear rate >1000× larger than used here), and it seems likely that these droplets will coalesce with free oil when the sample is on a roller bank^13^. It is possible that this process, as well as unbridging events, will lead to the release of free silica particles.

One possible explanation for the steadily increasing mixing of particle colours on the droplet surfaces is that these free silica particles are re-adsorbed onto different droplets. To test this hypothesis, we have conducted experiments where free red particles are added to the continuous phase of an emulsion which is stabilised by green particles and the samples are then placed on a roller bank. If the emulsion is initially bridged, then the free silica particles adsorb to droplet interfaces and the degree of bridging in the emulsion reduces, leaving the continuous phase clear of particles after 1 h of rolling, as shown in Fig. 5(e–g). Note that this is a very short disaggregation time compared to emulsions without added free particles. We conclude that any particles which desorb from an oil-water interface will generally re-adsorb to another droplet’s interface. The particle re-adsorption process is facilitated by the relatively large bare oil-water surface area in an emulsion with many bridges which are continually breaking and re-forming. However, the observation that clusters of droplets, which are held together by particle bridges, become mixed as the sample is rolled shows that particle transfer will occur during the unbridging/rebridging process, even in the absence of free silica particles. The increase in heterogeneity of the droplet surfaces cannot be explained by coalescence because the primary droplet size in the emulsion does not increase with time (see Fig. 2(a,b,e,f)). All droplets remain of the original size, which can happen because free silica particles are re-adsorbing to bare regions of oil-water interface, or alternatively they become part of the free oil layer.

The minor role played by coalescence is further emphasised by the fact that for almost all droplets it is easy to discern whether they are dominated by red or green particles (even when most droplets have become significantly heterogeneous as in Fig. 3(d)). Given that these droplets also only change colour slowly, this suggests that the cause of the heterogeneity is a gradual transfer of particles during the disaggregation process rather than coalescence. It can also be seen from normalised plots of the colour index, *f*, in Fig. 3(i) that the peaks in the distributions move inwards, and eventually meet at *f* = 0 (*f* varies from −1 for completely green droplets to +1 for completely red droplets; see Methods for calculation details). This trend is further evidence that the heterogeneity of the droplets is caused by gradual particle transfer processes rather than coalescence, since coalescence would be expected to generate a third peak at *f* = 0. We emphasise that this conclusion is strengthened by the fact that the droplets which are left at this stage have a mean size which is no larger than that of the mean primary droplet size of the initial emulsion (*i.e*., they are unlikely to have undergone coalescence). The behaviour of the particles at the droplet interface is summarised in Fig. 5(c).

### Weakening bridges via particle wettability

To test our understanding of this phenomena, an experimental run was carried out with *ϕ*_p_ = 0.6% and Φ_IM_ = 10% (see Fig. 2 (bottom row)). When the volume fraction of isopropyl myristate in the oil phase is reduced to 10%, the particles sit further into the aqueous phase (*i.e., θ*_w_ decreases), and the trapping energy of the particles at the oil-water interface decreases^13^. This means that the bridges are weaker, easier to break and less likely to re-form. It is therefore expected that the clusters in this sample will re-arrange more quickly and that disaggregation will occur sooner. Furthermore, when the particles are less strongly held at the interface, they are more likely to be released into the continuous phase during an unbridging event, and the increased concentration of free silica particles will further speed up disaggregation.

The sample with weaker bridges (Φ_IM_ = 10%) followed a similar behavioural pattern to the Φ_IM_ = 50% sample: both samples were initially dominated by bridged droplets, and remained so until the cumulative rolling time was at least 30 h. The Φ_IM_ = 50% sample does not fully disaggregate until the rolling time is at least 185 h (Fig. 2(b)), and possibly is not fully disaggregated after 357 h. However, the Φ_IM_ = 10% sample has fully disaggregated by 185 h of rolling. Following disaggregation, both samples are relatively stable, and although a layer of free oil develops and grows in volume throughout the experiment, this layer is never large enough to see in photographs. Quantitative analysis shows that the heterogeneity of the droplets in this sample follows a very similar trend to that of the Φ_IM_ = 50% sample, as shown in Fig. 3(f).

The most striking difference between the two samples during the period where they are both still strongly bridged is that the clusters of droplets in the Φ_IM_ = 50% sample are significantly less likely to become mixed, as can be seen in Fig. 2(g,h). It takes approximately 15 h of rolling for the clusters in the Φ_IM_ = 50% sample to reach a similar level of mixing to that of the Φ_IM_ = 10% sample after 1 h of mixing. The stronger particle bridges in the Φ_IM_ = 50% sample mean that the clusters are less likely to break up during the rolling process, and so the majority of a droplet’s neighbours continue to be from the same precursor emulsion as the droplet itself. In the Φ_IM_ = 10% sample, however, the weaker particle bridges are more likely to break during the rolling process, leading to the mixed clusters of droplets seen in Fig. 2(h). In addition to having stronger bridges, the sample with the higher isopropyl myristate content also experiences stronger particle-interface attractions, increasing the probability of rebridging and prolonging the lifetime of the bridged state.

### Removing bridges using salt

When a relatively low concentration of sodium chloride (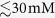) is added to the aqueous phase of the samples (composition: *ϕ*_p_ = 0.6%, Φ_IM_ = 10%), the particles’ contact angle decreases and the bridging behaviour is suppressed^13^. Nonetheless, particles are transferred between droplets when the samples are rolled, albeit at a greatly reduced rate, as shown in Figs 3(f) and 5(d). The main process driving droplet heterogeneity in this case is likely to be the re-adsorption of free silica particles from the continuous phase. However, it is also possible that particle transfer could occur during collisions between droplets, with temporary particle bridges forming and quickly being broken.

## Discussion

We have shown that particles are transferred between droplets in Pickering emulsions undergoing gentle shear, despite the large particle trapping energy, and that particle transfer occurs in all emulsions which have been studied here. The rate of particle transfer is greatest in bridged emulsions (*i.e*., where the droplets share particles), as these samples undergo a period of unbridging and rebridging prior to disaggregation. Particle transfer also occurs in samples which are not bridged, or which contain very few particle bridges, as shown by the sample with an aqueous phase sodium chloride concentration of 30 mM. However, the rate of transfer is greatly reduced in these samples. We suggest that the increase in droplet heterogeneity is driven by two main processes: particle transfer during unbridging/rebridging, and the re-adsorption of particles which have been released into the continuous phase. The particle transfer process is shown in the cartoons in Fig. 5(h–k), and dark spots which are thought to be caused by particle transfer are sometimes visible on droplets, as shown in Fig. 5(b). The particle re-adsorption process is depicted in Fig. 5(l–o). Coalescence events of the type depicted in Fig. 5(m) have previously been observed at low shear rates^13^. Under gentle rolling the coalesced droplets may rapidly become part of the free oil layer.

The disaggregation of bridged emulsions has been shown to be preceded by a relatively long period of unbridging and rebridging. The timescale of disaggregation is linked to the degree of bridging in the samples, and so can be tuned by varying the particle wettability or the shear history of the sample^13^. Since sudden disaggregation can also be induced by varying the particle wettability, (*e.g*., by dramatically changing the pH), a broad spectrum of disaggregation behaviours is accessible.

The application of low shear to emulsions which have been formed by mixing two strongly bridged precursor emulsions, each stabilised by a differently labelled batch of silica particles, has provided key insights into the disaggregation behaviour of bridged Pickering emulsions, as summarised in the cartoons in Figs 1(e) and 5(c). These experiments have shown that disaggregation is preceded by a relatively long period of unbridging and rebridging, during which substantial droplet rearrangement and particle transfer takes place.

The time taken to disaggregate is seen to be higher in the sample with the higher value of Φ_IM_, which we believe is due to the increased strength of the particle bridges in this sample, and so the disaggregation time can be tuned by modifying the particle wettability. Disaggregation is also probably accelerated in these experiments by a gradual decrease in contact angle due to the particles becoming more hydrophilic as they are immersed in water, but disaggregation should occur eventually without a change in wettability, as was seen in ref. 13.

We have demonstrated that the interfacial particles, which stabilise droplets in a Pickering emulsion, are not irreversibly adsorbed. Our results are consistent with the particles being prised off the interface during unbridging events. Here small forces can give rise to large torques due to the relatively large size of the droplets. During our experiments the bridging particles were being repeatedly ripped off one (or occasionally both) of the droplet interfaces to which they had previously been attached, before then being re-adsorbed elsewhere. Over a timescale of months, while this went on, a large proportion of the droplets remained stable and did not increase in size. This dynamic picture of Pickering emulsions is at complete variance to the existing literature on the subject^1^,^2^,^3^,^4^,^5^,^6^,^7^,^8^,^9^. As Pickering emulsions begin to be exploited, understanding at this level is crucial for controlling stability, encapsulation and flow properties.

## Methods

### Silica particles

Two differently dyed batches of Stöber silica particles have been used - green: chemically bonded via 3-(aminopropyl)triethoxysilane (APS) to fluorescein isothiocyanate (FITC), red: chemically bonded via APS to Rhodamine B isothiocyanate (RITC). In each experiment two separate precursor emulsions were created, one with each batch of particles, and these two emulsions were then gently mixed together and subjected to shear.

The particles used in this work were synthesised using a modified Stöber silica method to incorporate fluorescent dye^20^,^21^. The particles have radii of approximately 400–500 nm, are monodisperse, spherical, and charge-stabilised. The synthesis procedure used was as in ref. 22 for FITC-labelled particles and as in ref. 23 for RITC-labelled particles; in both cases APS is added in excess and is expected to have a dominant effect on the particles’ surface chemistry. The silica particles were washed ten times with distilled water to remove the ammonia, and then dried for one hour in a vacuum oven pre-heated to 170 °C, rendering the particles hydrophobic enough to sequester to the oil–water interface^24^.

### Particle dispersions

Aqueous dispersions of silica particles were prepared as follows. Particles were dried under vacuum at 170 °C for one hour and were then added to 2.33 mL of water (deionised using a Millipore Milli-Q reagent system; resistivity of at least 18.2 MΩ cm) and dispersed using a pulsed ultrasonic probe (Sonics Vibracell). A typical dispersion protocol involved 1 s of sonication followed by 5 s of cooling, repeated for a total time of 6 min. 0.67 mL of the oil phase, a mixture of dodecane (Sigma-Aldrich, ≥99% - filtered twice through alumina) and isopropyl myristate (Sigma-Aldrich, ≥98% - used as received), was then added prior to the initial emulsification. Interfacial tensions for mixtures of dodecane and isopropyl myristate, and experimentally measured contact angles for this system, are reported in ref. 13. The volume fraction, Φ_IM_, of isopropyl myristate in the oil phase is used to control the contact angle^17^. The particle density was assumed to be 1750 kg m^−3^, when calculating particle volume fractions, *ϕ*_p_.

### Emulsion formation

Emulsification of the precursor samples was carried out by vortex mixing the samples in 30 s bursts until the aqueous phase was clear of particles. Subsequently, the precursor emulsions were combined - with a small amount of water being added to the resulting two-colour sample as part of washing out the vials used to emulsify the initial emulsions. The resulting two-colour sample was then placed on a roller bank (Stuart Equipment SRT9) for varying periods of time.

### Particle re-adsorption experiments

In the particle re-adsorption experiments, initial emulsions were created as above, with *ϕ*_p_ = 0.6% and a sample volume of 3.0 mL. 0.5 mL of a particle dispersion with *ϕ*_p_ = 0.75% was then added to each sample before the sample was placed on a roller bank.

### Sample characterisation

Confocal microscopy, bright-field microscopy, photography and light scattering were used to characterise the emulsified samples. A Beckman Coulter LS 13 320 Particle Size Analyzer was used to measure the apparent size distributions of emulsions as they disaggregate. This method is only accurate when the droplets are not bridged; when the droplets are bridged, light scattering can provide a quantitative measure of the degree of bridging. It should be noted that there are several problems which must be accounted for when using light scattering to measure the apparent size distributions of disaggregating emulsions^25^. For this reason, comparisons with photography and bright-field microscopy have been used to corroborate the light scattering data. In most bridged samples, the fraction of the emulsion which was incorporated into a cluster could be estimated by the area under the bridging peak of the apparent size distributions.

Samples were prepared for microscopy by pipetting a drop of the emulsion onto a glass cavity slide, diluting it in its continuous phase and covering with a glass coverslip. Epoxy resin was used to seal the slides in order to prevent evaporation and flow.

A Qimaging QICAM Fast 1394 monochromatic camera, attached to an Olympus BX50 optical microscope, was used to obtain bright-field micrographs of emulsions.

A Zeiss LSM 700 confocal microscope was used to qualitatively and quantitatively measure both the colour heterogeneity of individual droplets and the mixedness of clusters of bridged droplets. A 10 mW, 488 nm solid-state laser was used to excite particles dyed with FITC and a 10 mW, 555 nm solid-state laser was used to excite particles dyed with RITC. Generally, the confocal microscope was operated using a 10× objective lens with a numerical aperture of 0.3, which is just powerful enough to resolve individual particles, but gives a large field of view. High magnification confocal micrographs were taken using a 63× oil immersion lens with a numerical aperture of 1.3.

### Quantitative image analysis

Quantitative analysis of the confocal micrographs was carried out using MATLAB^26^ and GNU Octave^27^. Briefly, the intensities of the red and green channels (*I*_R_ and *I*_G_, respectively) are smoothed slightly, with a circular uniform filter of 5 pixels radius, and normalised by multiplying *I*_R_ by a factor of 〈*I*_G_〉/〈*I*_R_〉. The following value was then calculated for each pixel:


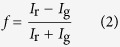


where *I*_r_ and *I*_g_ are the smoothed, normalised intensities of the red and green channels, respectively. The calculation is also performed for those pixels at the centres of droplets (identified either using a threshold or manually), and the mean absolute value for each image is denoted 〈|*f*_d_|〉. In order to exclude background noise, pixels with a combined intensity below a threshold value are excluded from the calculations. Histograms are created showing the number of droplets, *N*, with values of *f* which fall into each of 10 equally sized bins. Normalised line plots are also shown, which show the fraction, *N*′, of pixels with *f* values in each of 100 equally sized bins.

### Electron microscopy

Freeze-fracture scanning electron microscopy (FFSEM) was carried out using a Hitachi S-4700 field emission scanning electron microscope, as described in ref. 13.

## Additional Information

**How to cite this article**: French, D. J. *et al*. The secret life of Pickering emulsions: particle exchange revealed using two colours of particle. *Sci. Rep*. **6**, 31401; doi: 10.1038/srep31401 (2016).
